# Increasing Accuracy: A New Design and Algorithm for Automatically Measuring Weights, Travel Direction and Radio Frequency Identification (RFID) of Penguins

**DOI:** 10.1371/journal.pone.0126292

**Published:** 2015-04-20

**Authors:** Vsevolod Afanasyev, Sergey V. Buldyrev, Michael J. Dunn, Jeremy Robst, Mark Preston, Steve F. Bremner, Dirk R. Briggs, Ruth Brown, Stacey Adlard, Helen J. Peat

**Affiliations:** 1 British Antarctic Survey, Natural Environment Research Council, High Cross, Madingley Road, Cambridge, CB30ET, United Kingdom; 2 Department of Physics, Yeshiva University, 500 W 185th Street, New York, NY, 10033, United States of America; University of Lincoln, UNITED KINGDOM

## Abstract

A fully automated weighbridge using a new algorithm and mechanics integrated with a Radio Frequency Identification System is described. It is currently in use collecting data on Macaroni penguins (*Eudyptes chrysolophus*) at Bird Island, South Georgia. The technology allows researchers to collect very large, highly accurate datasets of both penguin weight and direction of their travel into or out of a breeding colony, providing important contributory information to help understand penguin breeding success, reproductive output and availability of prey. Reliable discrimination between single and multiple penguin crossings is demonstrated. Passive radio frequency tags implanted into penguins allow researchers to match weight and trip direction to individual birds. Low unit and operation costs, low maintenance needs, simple operator requirements and accurate time stamping of every record are all important features of this type of weighbridge, as is its proven ability to operate 24 hours a day throughout a breeding season, regardless of temperature or weather conditions. Users are able to define required levels of accuracy by adjusting filters and raw data are automatically recorded and stored allowing for a range of processing options. This paper presents the underlying principles, design specification and system description, provides evidence of the weighbridge’s accurate performance and demonstrates how its design is a significant improvement on existing systems.

## Introduction

Routinely monitoring the body weight and foraging trip duration of individual adult penguins and other marine birds is a powerful research tool. It provides information on the availability of prey species in the ocean [1–6], and allows regular monitoring of chick meal sizes which is an important factor contributing towards breeding success and reproductive output [7–9]. In this paper we describe a reliable and highly accurate method of measuring a large number of penguin weights throughout the breeding season. These data can be used to calculate 1) chick meal size, 2) parental weight change during any given breeding season for a large number of individual adult penguins, and 3) foraging trip number and duration.

Since all penguins are obligate marine foragers, during the breeding season adult penguins, regardless of species, regularly leave their colony/nest site to forage at sea, returning with food in their stomachs in order to provision their chicks [9]. The difference in weight of an individual adult penguin between returning to, and leaving its breeding colony to forage at sea, is considered to be a reasonable proxy of chick meal size [1,7]. Manually weighing penguins, however, is very labour intensive and involves high levels of disturbance as each bird weighed has to be captured twice [10,11]. The accuracy of data collected in this way is also limited by the accuracy of the manual weighing equipment used. Consequently, collecting large amounts of data in this way is impractical and causes stress and disturbance to birds in the study colony [12]. A better solution is to automatically log information related to the weight of individual penguins over an extended, continuous period, such as an entire breeding season. Automated systems capable of recording the identity of individual birds, together with their direction of travel as they pass over an instrument placed between their breeding colony and the sea, greatly reduces the need to handle birds and is therefore particularly beneficial when data are required on a regular basis. An automated system of this type was first described in 1993 by Kerry et al. [13] to study Adélie penguins. Since then a number of research groups have successfully developed their own systems [1,7,13–18] to monitor the weights of a variety of penguin species, for example Adélie penguins (*Pygoscelis adeliae*) at Béchervaise Island [13,15] and Cape Crozier [14,16], Southern Rockhopper penguins (*Eudyptes chrysocome*) at New Island, Falkland Islands [18] and Little penguins (*Eudyptula minor*) at Phillip Island, Australia [17]. These weighbridge systems use internal empirical processing algorithms to generate a penguin mass which is then adjusted following a comparison of the weighbridge masses with masses derived using a spring dynamometer. The raw data used by the algorithms are not retained. The accuracy of existing systems is limited due to:

- low sampling frequencies,
- empirical algorithms using arbitrary cut-off values to judge when more than one penguin is present on the bridge,
- infrequent calculation of the offset
- a lack of clarity as to how to deal with weights of penguins when a bird is half on and half off the bridge
- no account being taken of the velocity of the penguin in addition to the mass

It is also impossible to judge how well the empirical processing algorithms work as the raw data are not stored. Our system was developed to overcome these accuracy issues. We use sound physical laws to explicitly explain how penguin weights are calculated, utilising both mass and velocity (previously reported systems rely solely on mass, [17,18]). The system we have designed stores all the forces generated by a penguin as it crosses a weighbridge platform. Retention of the raw data makes it possible to test different data processing algorithms, to check the accuracy of these algorithms and to examine data manually. A description of a system like this has not been published before.

This paper describes the theory behind using force records as a penguin crosses a weighbridge to calculate penguin weight; the design of a weighbridge which can do this to user defined levels of accuracy; the deployment of the equipment in a penguin colony; and the subsequent processing of data to calculate penguin weight and direction of travel. This study was carried out following approval by the British Antarctic Survey Animal Ethics Review Board. No animals were harmed. In 2013/14 permission to work at Bird Island, South Georgia was given in Scientific Permit SCI/2013/005 from the Government of South Georgia and the South Sandwich Islands. This is the first year of issue of permits for scientific work at this location, prior to this a written letter of authority was provided by the Office of the Commissioner, Government House, Stanley, Falkland Islands, to British Antarctic Survey. No endangered or protected species were involved in the study. The study site is at 54.0105 degrees South, 38.0738 degrees West.

### Theoretical rationale behind the weighbridge approach

We have developed an efficient and accurate *ab initio* algorithm based on published physical laws, eliminating the need for our own empirical models.

A penguin walking on a weigh bridge is acted upon by three forces: its weight, reaction of the bridge and air resistance. According to Newton’s Third Law the vertical component of the bridge reaction equals the recorded force *F*(*t*), where *t* is time. Neglecting air resistance we can then write Newton’s Second Law in the integral form:

$$m\left[v\left(t_{2}\right)-\;v\left(t_{1}\right)\right]=\;\int_{t_{1}}^{t_{2}}\left[F\left(t\right)-\;F_{0}-\;mg\right]dt$$ (1)

where *m* is the unknown mass of the penguin; *t*_2_ *> t*_1_ are two arbitrary moments of time; *v*(*t*) is the vertical component of velocity of penguin’s centre of mass; *F*(*t*) is the force measured by the bridge; *F*_0_ is the force measured by the bridge when the bridge is empty; and *g* ≈ 9.8m/s^2^ is Earth’s gravity constant [19].

Solving Eq (1) for weight *mg*, we find:

$$mg=\frac{\int_{t_{1}}^{t_{2}}\left[F\left(t\right)-F_{0}\right]dt}{t_{2}-t_{1}}\;-\;m\frac{v\left(t_{2}\right)-v\left(t_{1}\right)}{t_{2}-t_{1}}$$ (2)

The first term on the right-hand side of Eq (2) is the mean weighbridge output between user selectable limits *t*_1_ and *t*_2_ (Mean Value Theorem) and is straightforward to calculate. We propose to use this mean as a first approximation of the static weight of a penguin,

$$mg\;\approx \;\frac{\int_{t_{1}}^{t_{2}}\left[F\left(t\right)-F_{0}\right]dt}{t_{2}-t_{1}}$$ (3)

The second term on the right-hand side of Eq (2),

$$m\frac{v\left(t_{2}\right)-v\left(t_{1}\right)}{t_{2}-t_{1}}$$ (4)

is an error associated with the first approximation (3).

In many cases, a careful selection of the limits of integration *t*_*1*_ and *t*_*2*_ allows us to make this error acceptably small or even negligible:

- Firstly, the length of the integration interval appears in the denominator of the error (4). This means that the longer the integration interval the higher the accuracy.
- Secondly, if the steps of the penguin are repetitive (and very often they are), the functions *F*(*t*) and *v*(*t*) are both quasi-periodic, and it is possible to identify the moments of time *t*_1_ and *t*_2_ when the steps enter the same phase. For these moments the *v*(*t*) values are roughly the same, that is, the numerator *v*(*t*_2_)*—v*(*t*_1_) of the error (4) is small. Such moments of time are the best points to start and end the integration of the recorded signal. Therefore, we have the second criterion of record quality: the more repetitive the records, the higher the accuracy of the first approximation.

There is a situation in which the estimated error can approach zero, e.g. if a penguin stops on the bridge. During this interval of time the vertical component of velocity of its centre of mass is zero and therefore, choosing *t*_1_ and *t*_2_ at the start and end of this interval assures the zero error term (4).

First approximation of penguin weight (Mean Value Theorem):

$$W_{1}=\frac{\int_{t_{1}}^{t_{2}}\left[F\left(t\right)-F_{0}\right]dt}{t_{2}-t_{1}}\equiv \bar{F}-F_{0}$$ (5)

where *W*_1_ is the first approximation of penguin weight, $\bar{F}$ meaning arithmetic mean of *F* during time interval from *t*_1_ to *t*_2_.

**Fig 1:**
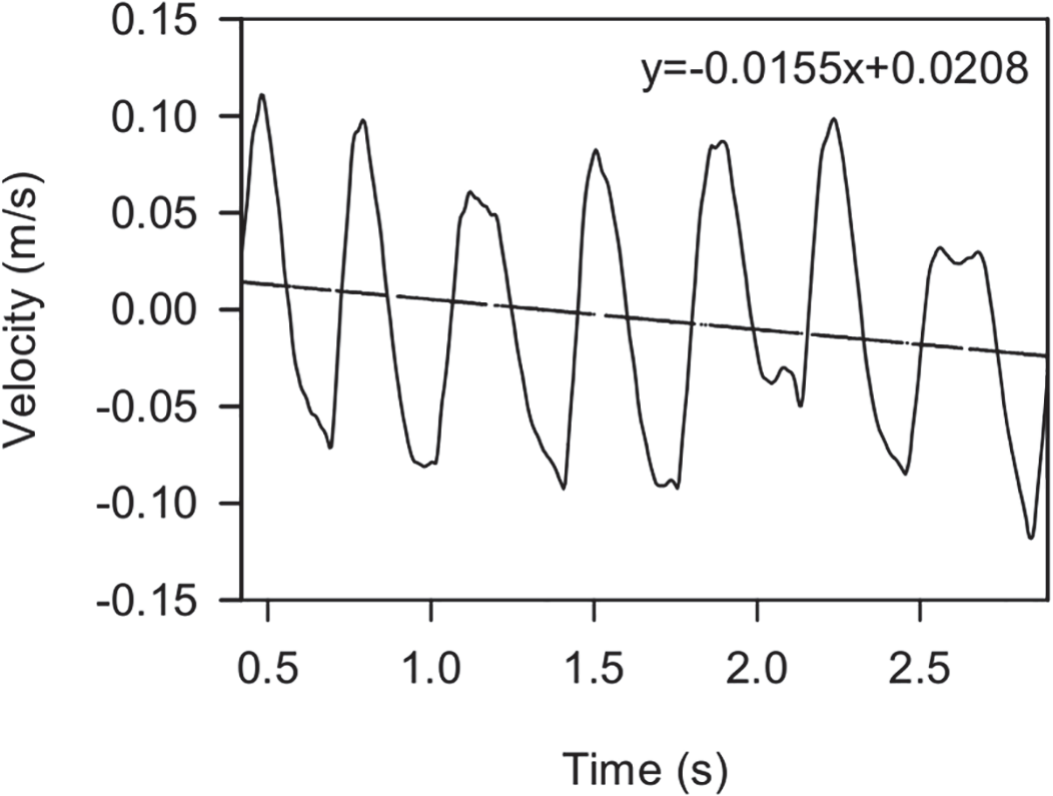
Vertical component of the velocity of the centre of gravity of the penguin. The figure shows the vertical component of the velocity of the centre of gravity of the penguin *v*(*t*), calculated using the first approximation (6). Any velocity trend (slope) indicates the weight estimate is in error. The slope of the linear regression (also shown) gives the relative error of the first approximation (8). Thus we calculate the second approximation of the penguin weight (10).

Though we do not make any attempt to actually measure *v*(*t*_1_) or *v*(*t*_2_) it is in fact possible to estimate the values *v*(*t*) and so to assess the error (4) associated with the approximation (3) in any particular case from just a force record. Assuming *m* = *W*_1_ / *g* obtained from the first approximation and substituting it into Eq (1) written for an arbitrary upper limit of integration *t*, we get:

$$v\left(t\right)=v\left(t_{1}\right)+\;\frac{g}{W_{1}}\;\int_{t_{1}}^{t}\left[F\left(t\right)-F_{0}-W_{1}\right]\;dt,$$ (6)

where we expect *v*(*t*) to oscillate about 0. Any velocity trend (slope) indicates that the weight estimate is in error (Fig 1). To calculate it we use the linear least squares fitting technique:

$$a\;=\;\frac{\sum_{t_{1}}^{t_{2}}\left(t_{i}-\bar{t}\right)\left(\Delta v_{i}-\bar{\Delta v}\right)}{\sum_{t_{1}}^{t_{2}}\left(t_{i}-\bar{t}\right){\;}^{2}}$$ (7)

where *a* is the trend (slope) of linear regression of *v*(*t*), *t*_*i*_ are the equidistant moments of time at which the measurements were done *t*_*1*_ *≤ t*_*i*_ *≤t*_*2*,_ and Δ*v*_*i*_ = *v*(*t*_*i*_)−*v*(*t*_1_) are computed using Eq(6). Subtracting linear function *at* from the right hand side of Eq (6) eliminates the trend. Thus the value *a/g* gives the relative error of first approximation:

$$\frac{\Delta W}{W_{1}}\;=\;\frac{a}{g},$$ (8)

where *ΔW* is absolute error of *W*_1_. Accordingly, the second approximation *W*_2_ of penguin weight is

$$W_{2}=W_{1}+\;\Delta W=\;W_{1}+\;W_{1}\frac{a}{g}$$ (9)

or

$$W_{2}=\;W_{1}\;\left(1+\frac{a}{g}\right).$$ (10)

This second approximation makes the method less sensitive to selection of the limits of integration *t*_1_ and *t*_2,_ and provides a more accurate value of the penguin weight, particularly in cases where less than optimum limits of integration were chosen. We estimate the accuracy of the second approximation to be 0.1% or ±4g.

### System requirements

The weighbridge was designed to meet the following requirements:

- Daily 24 hour coverage throughout the breeding season.
- Sufficient accuracy—the aim was for 1% or ±40 g.
- High resonant frequency. Penguin movements should not force the system to oscillate.
- Sampling rate satisfactorily above resonant frequency. To avoid distortions, the sampling rate *Δt* must exceed the Nyquist rate [20]. We estimate that *Δt* = 200s^-1^ is adequate.
- Sufficient range—the landing force of a hopping penguin is much greater than penguin weight.
- Accurate time stamping of every record. (Recording period is 7 months).
- Reliable discrimination between single and multiple bird crossings.
- Reliable detection of the identity of birds crossing the weighbridge.
- Retention of all raw data to allow for a range of processing options and for users to apply different data validity criteria depending on their research objectives.
- A nonconductive platform. The system includes a radio tag reader (RF ID), so it was essential that parts of the systems do not interfere with one another.
- Low power consumption. It should be able to be operated from a manageable size solar power supply for several months.
- High levels of environmental protection. The system needs to withstand frequent freezing and thawing conditions, salt water spray from the sea, wind, rain, snow and mud. No keyboard input should be required at the colony.
- The system should be user friendly and very simple to mobilise, operate and demobilise. No technical support should be required.

### Location

**Fig 2:**
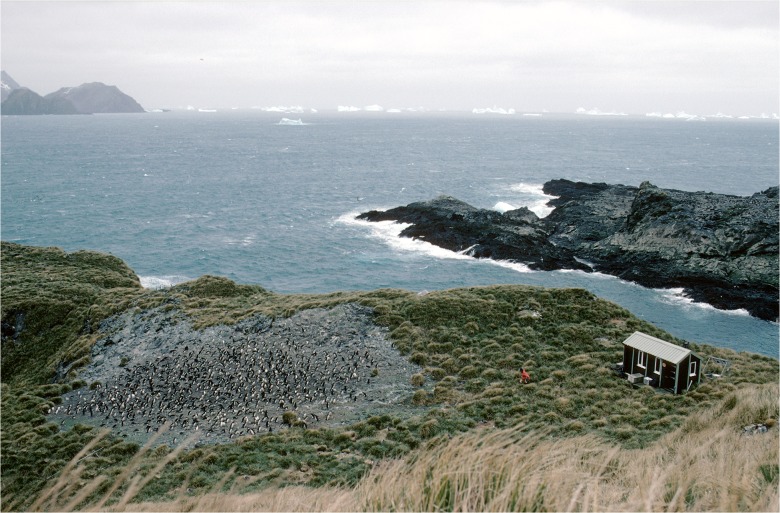
Photograph showing the weighbridge location. The terrain around the colony is such that there is only one route penguins can take both on their approach to the colony and on their return to the sea.

The weighbridge was deployed next to a relatively small breeding colony (approximately 400 pairs) of macaroni penguins (*Eudyptes chrysolophus*), at Bird Island, South Georgia (54°00' S, 38°03' W). Monitoring of macaroni penguins has taken place at this locality (Fig 2) since 1982 and submitted to the Commission for the Conservation of Antarctic Marine Living Resources (CCAMLR) Ecosystems Monitoring Programme (CEMP) since 1989. A total of 2375 penguins from this colony have been implanted with radio frequency identification transponders (RFID tags) since 2003; an average of approximately 100 adults and 100 chicks receiving implants under their skin each year following standard methodology [10,13,16,20,21]. The terrain around the colony is such that there is only one route penguins can take both on their approach to the colony and on their return to the sea. A gateway, containing the weighbridge was built on this route to ensure that penguins have to cross the weighbridge when both leaving, and returning to, the colony.

Initial trials of the weighbridge were undertaken in 2008–2009. The weighbridge has been deployed throughout the breeding season in every following year. In 2009/10 and 2010/11 penguin IDs recorded by RFID antennae were collected by a completely separate system [22]. From 2011/12 onwards the RFID antenna was built into the weighbridge, as described below, making it possible to confidently match the crossing data with the identification data.

### System description

**Fig 3:**
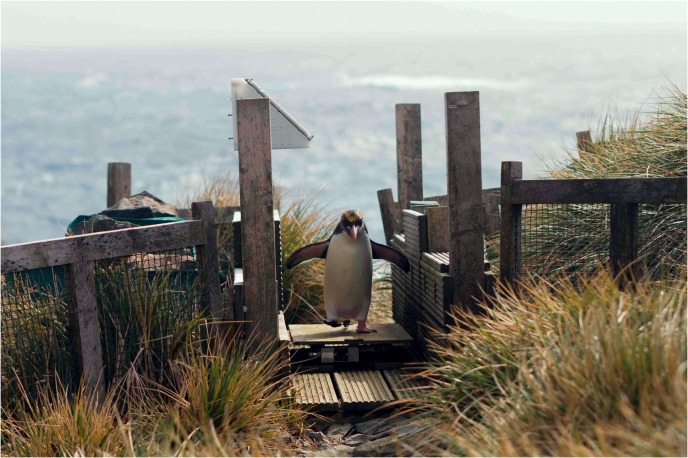
Photograph of a penguin crossing the weighbridge.

The weighbridge is two horizontal rectangular “marine plywood” panels one above the other. This is specially treated at manufacture to resist distortion in a high-moisture environment. The bottom panel rests on the ground. For stability the panel is heavy 18mm “marine plywood”. The top panel is thinner 9mm “marine plywood”. This is for penguins to walk on (Fig 3). The panels are separated by two load cells, one cell at each end of the bridge.

We used type PC6 Load Cells from FLINTEC with maximum capacity of 50 kg and ultimate load of 150 kg each. Such heavy ratings were required to prevent possible damage by fur seals (*Arctocephalus gazella*) The compensated temperature range of the load cells is—10°C / +40°C and they have complete hermetic sealing to IP 68 (“protection against indefinite submersion in water under pressure at one meter depth”) to prevent water ingress.

A Texas Instruments RFID antenna is also bolted to the bottom panel on nonconductive spacers. It is located just under the top panel. Connected to the RFID antenna is a TIRIS reader S251B from Texas Instruments.

**Fig 4:**
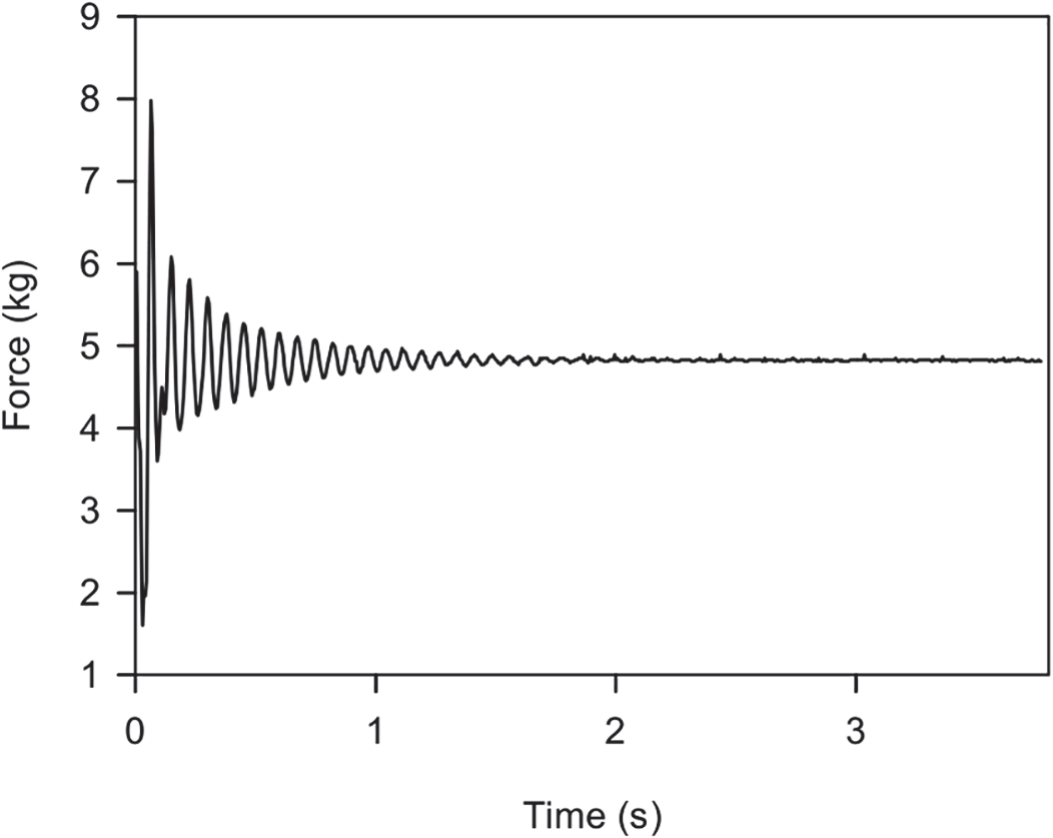
Graph showing the oscillation of the weighbridge. This graphs shows the oscillation when a 4 kg weight is used for calibration. The high resonant frequency of the system ensures that penguin movements do not force the weighbridge to oscillate at resonant frequency. The top panel oscillating in air at this frequency has high drag which rapidly dampens the oscillation. As a result the system quickly (in a couple of seconds) stabilises and data can be used for calibration.

Since the top panel rests on the load cells the bridge acts as a mechanical oscillator. Therefore, it is designed so that the penguin movements do not force it to oscillate at a resonant frequency. High capacity load cells and a low weight top panel result in a high resonant frequency (Fig 4) which reduces distortion and ensures a high level of accuracy. The current top panel is 800mm long, 400mm wide. The optimal length is a compromise between choosing a long length to increase the time each penguin remains on it (enabling a more accurate weight calculation) and a shorter length to reduce the likelihood of it being occupied by more than one penguin. If required, the length can be easily altered. The gateway was designed to be narrow enough to allow one penguin through at a time, without being so small that penguins were reluctant to travel across it. The weighbridge platform width was designed to fill the width of the RFID identification gateway. The weighbridge platform is set 95 mm above the surrounding ground to improve accuracy by preventing penguins from spreading their weight by standing both on the platform and surrounding ground at the same time.

Outputs of both load cells are fed into a purpose built analogue summing device. The output voltage of the summing device is a linear function of force applied to the platform and is independent of the point of its application. Offset voltage and gain are set independently in the hardware. The gain determines the range of the instrument and is currently set to just under 10 kg. Any difference between load cells and amplifiers can be cancelled out with manual adjustment of electronics at the manufacturing stage. Digitization resolution of this main channel is 9 bits. These data comprise the wgt1 data stream and are used for calculations of penguin weight.

The output of one of the load cells is recorded separately with a digitization resolution of 7 bits. This auxiliary signal depends strongly on the point of application of the force. At present, the platform is oriented in such a way that the auxiliary signal is generated by the cell at the colony side of the system. Consequently, if a force is applied at that end the auxiliary signal is larger than the main one. If a force is applied at the seaward side of the system, the auxiliary signal is smaller than the main one. These data comprise the wgt2 data stream and are used to establish the direction of travel of a penguin. In addition this stream is very useful for separating single penguin crossings from multiple penguin crossings. The weighbridge is powered by a 12 Volt sealed lead-acid battery Sonnenschein A512/40 with 40 Ah capacity and 14.5 kg weight. Battery life of the system is three months without recharging. To enable the system to run throughout the season the battery is recharged using a small solar panel (2.9 W, 280 mm by 160 mm). The RFID part of the system is powered by two 40 watt solar panels. Each panel has its own storage lead acid battery and an off- the- shelf charge regulator PR8 from PELANGI INTERNATIONAL LTD. All data collection and system control electronics were developed by engineers at the British Antarctic Survey (Natural Environment Research Council, Madingley Road, Cambridge, CB3 0ET).

**Fig 5:**
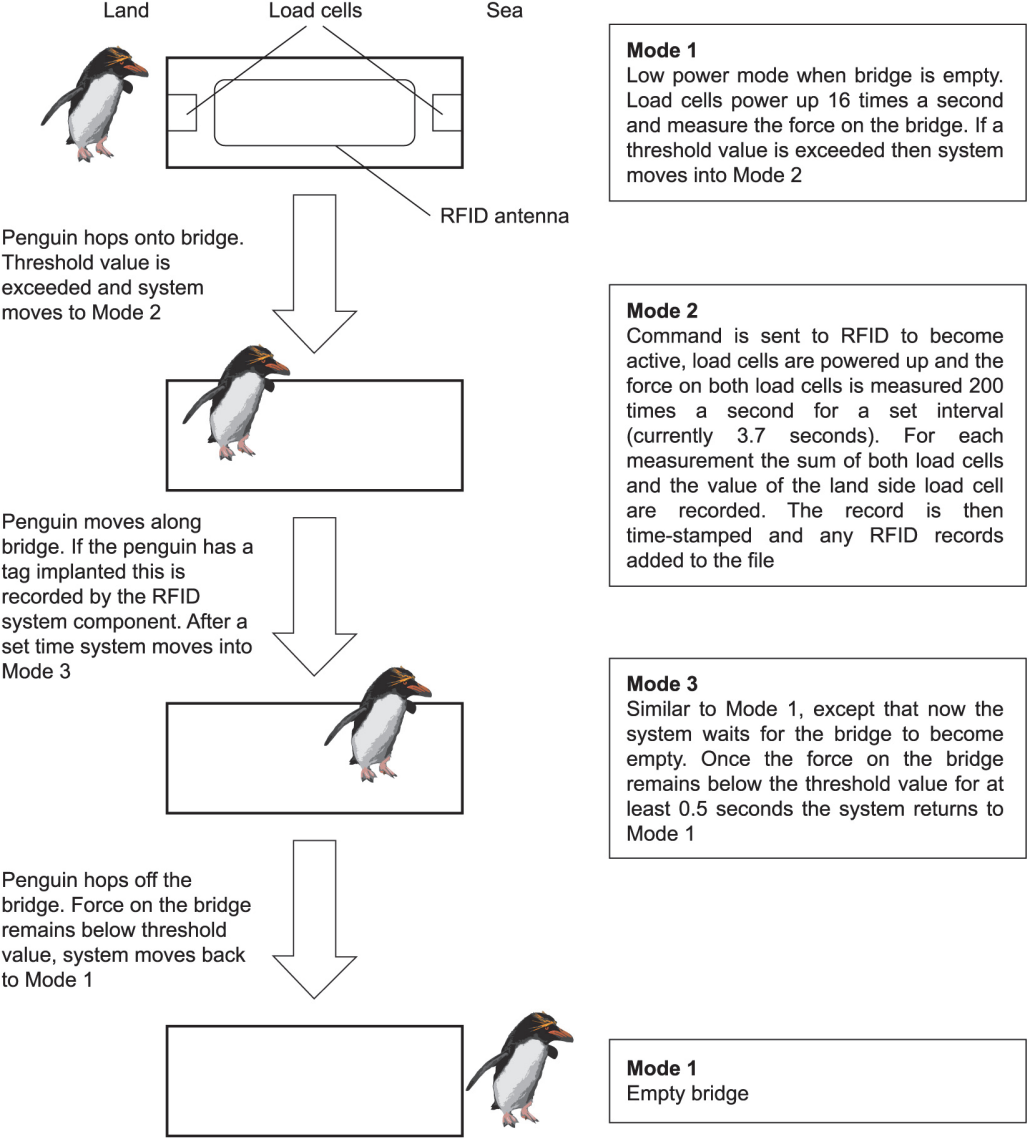
A flow diagram outlining how the weighbridge operates.

The system has three modes of operation which are described in Fig 5. Data recorded when the system is in mode 2 comprise the readings from wgt1 and wgt2, RFID records and a time stamp. These are stored in a flash memory digital logger. For memory and power saving, we use sampling rate Δ*t* = 0.0625s during modes 1 and 3, and Δ*t* = 0.005s during mode 2.

### Operating the weighbridge

The system is maintained by a field assistant who carries out calibration and data recovery procedures approximately once a week. To calibrate the system we used two 2 kg standard weights and one 5 kg standard weight. Combinations of standard weights give forces of 2 kg, 4 kg, 5 kg, 7 kg and 9 kg. This covers the full range of the instrument which is set to approximately 10 kg. Using standard weights in kilograms allows the forces applied to the weighbridge to be expressed as kilograms. Each standard weight combination in turn is placed on the top of the load cell at the seaward side of the colony followed by the top of the load cell on the landward side of the colony and the date and time of each weight placement recorded. Recording the date and time is important as it allows the calibration data files to be easily identified. Frequent calibrations are an important measure of system performance particularly to ensure that the relationship between forces applied to the top of the bridge and the voltages measured (the gain) remains stable.

For data recovery there are two identical waterproof plastic boxes containing flash memory digital loggers. One box is connected to the system and the other box is kept at the research station. At the time of data recovery the boxes are simply swapped. This takes 1–2 minutes and the size (180x180x82mm) and weight (0.7kg) of the boxes allows for easy transfer to and from the research station in a backpack. Swapping over the data box, users press the bridge and write down the time. This is the time of the first event of the next deployment. At the research station, data are transferred from the data box to a PC using a standard serial port and then deleted from the card in the data box.

At the end of each system visit both load cells are visually inspected for possible mud/debris accumulation and cleaned when necessary. Approximately ten minutes per week is required for system maintenance, calibration and data recovery.

### Data analysis

The raw data from the data box is downloaded via the XMODEM protocol using the standard Windows utility HyperTerminal, creating a binary data file. A small custom Windows application was written by the in house IT department to convert this file into Comma Separated Variable (CSV) files readable by many common processing applications. The conversion utility outputs one CSV file for each bridge crossing event detected by the system, and one CSV file for any RFID tags detected during the crossing event. Files are named according to the time the event took place. Since the system does not have a real time clock times are relative to when the system was powered up. The conversion utility can optionally correct the timestamps stored with each event to real time based on input from the user of either the time of the first event, the time of the last event or both times. Since a calibration is performed at the end of each weekly deployment these times are known. If both times are known the conversion utility will correct for any drift of the system's internal clock.

### Processing the downloaded data

All the data are loaded into an Oracle database and stored within the Polar Data Centre at the British Antarctic Survey. The RFID data are loaded into a table containing columns for the time stamp and tag numbers recorded and the weight data are loaded into a table with columns for the timestamp, the time unit of the force measured and the values for wgt1 and wgt2 where wgt1 is the sum of the two load cells and wgt2 is the value of the load cell on the land side of the weighbridge. For each weighbridge crossing that included a period when the weighbridge was empty, an offset value *F*_0_ was calculated for the crossing. For crossings which did not include a period when the bridge was empty, the nearest available offset value was assigned to that crossing. The offset value helps ensure that penguin weights are not over-estimated due to build up of dirt, snow or water on the weighbridge platform. Penguin weights are calculated by taking a mean of the forces measured between a peak towards the beginning of the crossing and a peak towards the end of the crossing as outlined below.

### Calculating penguin weight

1. A query was run to identify all peaks for each crossing, by chopping the data from each crossing into bins of 0.2 s and looking for the maximum force value in each bin. 0.2 s was chosen as the bin size as it approximated the time taken for most penguins to take one step. To avoid identifying false peaks at the bin boundaries, a peak was only counted if it was still identified as a peak when the bin boundaries were shifted by 0.025 s in either direction.
2. Peaks with a value below a set level indicating the bridge was empty were identified and this and all subsequent peaks were removed as they indicated the penguin had left the bridge. Deleting all subsequent peaks ensured that even if a second penguin entered the bridge only force values for the first penguin would be used to calculate a weight.
3. Peaks with a value above a set level, indicating there was more than one penguin on the bridge, were identified and the first and any subsequent peaks were removed to ensure weights were only calculated when a single penguin was present.
4. The average force on the weighbridge was then calculated for all time units between the time of the second and second to last peak—the first and last peak were removed as extra large forces tend to be generated when a penguin hops on and off the bridge.
5. If possible an offset value *F*_0_ for when the bridge was empty was calculated as an average of at least 0.25 s where wgt1 was below a set value (chosen by examining some of the graphs) and, in addition, there was little difference between the value of wgt 1 and wgt 2 (this indicates no movement across the bridge, helping confirm it is empty). This offset value was subtracted from the average force or, if there was no period when the bridge was empty, then the nearest available offset value prior to this crossing was used.
6. Force values were then translated into the first weight approximation in kilograms by dividing the average force by the calibration value. The calibration value was calculated as the slope of a linear regression of all the weekly calibration events.
7. The more accurate second weight approximation was then calculated using Eq (7) and (10) above.

**Fig 6:**
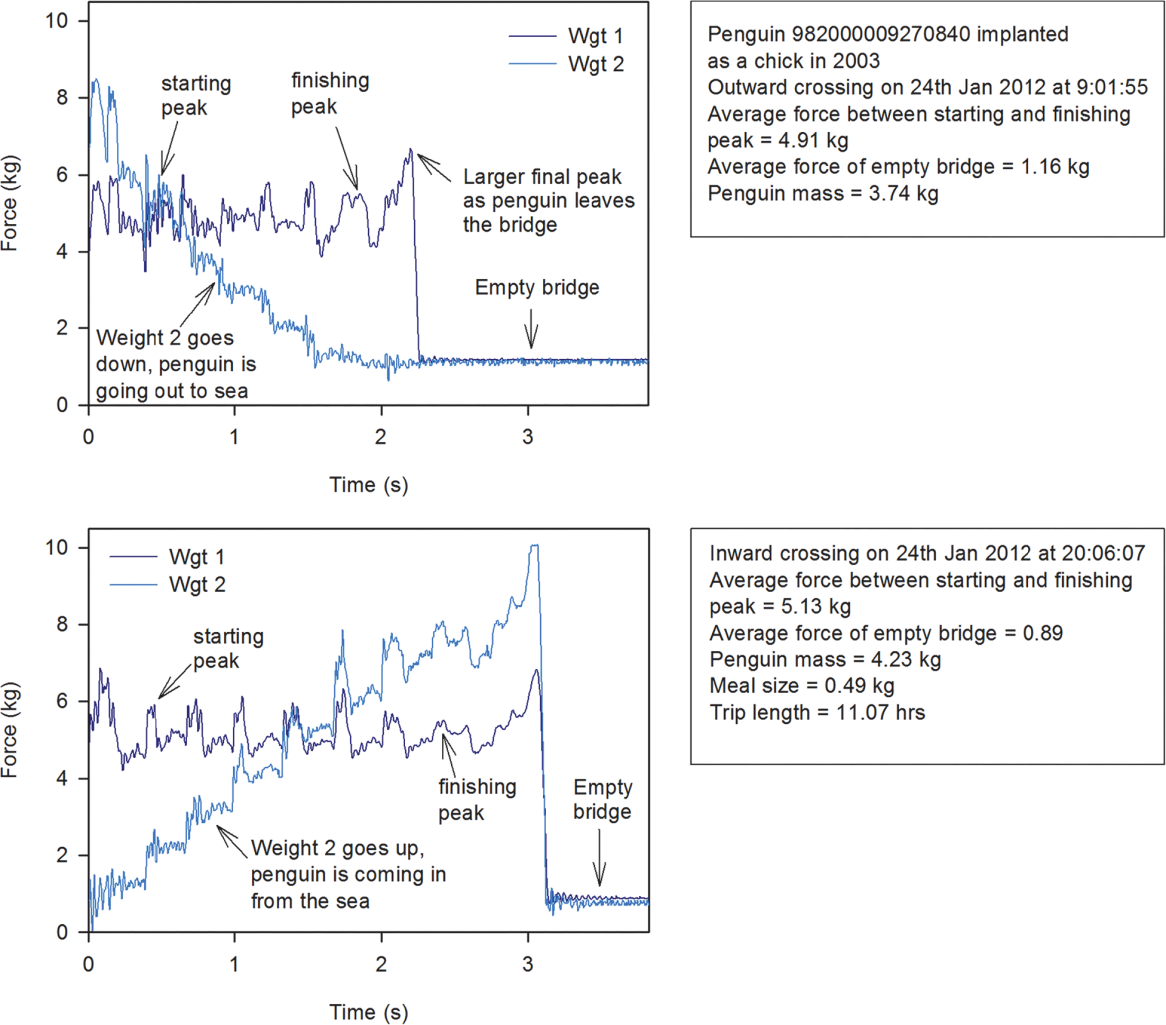
Penguin crossing graphs. Graphs showing both the outward morning crossing and the inward evening crossing of the same penguin when chick rearing.

**Fig 7:**
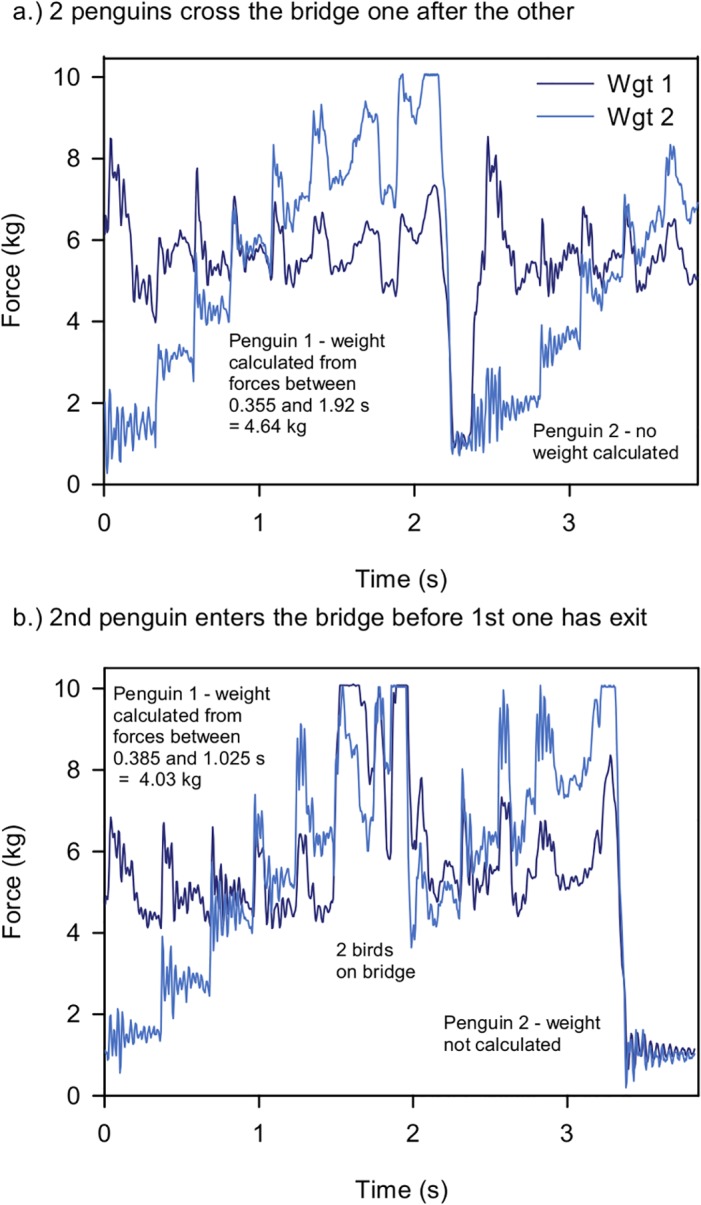
Multiple crossing graphs. Two weighbridge crossing graphs showing in a. one bird crossing the bridge, followed by a second bird entering the bridge. This second bird is still on the bridge when the weighbridge stops recording; and in b. one bird crossing the bridge and, whilst it is crossing, it is joined by a second bird. The first bird leaves the bridge, followed by the second bird leaving the bridge empty.

An ideal record for a single bird crossing the weighbridge will consist of a long duration when the penguin is on the bridge followed by a short period when the bridge is empty. Two typical single bird crossing files showing a known penguin leaving the colony in the morning and returning in the evening are shown in Fig 6. When two ideal crossings exist for the same bird on the same day during the chick rearing period, it is possible to calculate both trip length and meal size. The greater the time period between the two selected moments of time, the greater confidence can be placed in the calculated weight values. It is possible to select which weight values to use in any analyses depending on the length of time used for the weight calculation—for example during the 2011–2012 breeding season, weights were calculated for 58888 bridge crossings, with 76% having weights calculated from at least 1 s on the bridge, 54% from 1.5 s, 32% from 2 s and 19% from 2.5 s. Some records are less than ideal and files can contain more than one penguin which may be on the bridge simultaneously or one may enter when the first one has left; two examples are shown in Fig 7.

### Determining direction

The direction of the crossing was determined using two methods. Firstly, the value of wgt2 at the time of the second to last peak was subtracted from the value of wgt 2 at the time of the second peak. Positive values of over a set value (currently set at 60), were assigned as the penguin going out and negative values of less than the set value (-60) as the penguin coming in. Other values were assigned as ‘unknown’. Secondly, the mean value of wgt2 for the first 0.25 s was calculated. If this was less than a set value (currently 200) it was assumed that the penguin entered the bridge on the sea side (coming in) or if this was above a set value (currently 300) it was assumed that the penguin entered the bridge from the land side (going out). Other values were assigned a direction of ‘unknown’. High confidence could be attached to records where both methods agreed on direction. The small number of records with ‘unknown’ values, or those with disagreement between the two methods could either be examined to assign the correct value or be discarded from any analyses. Unknown values or disagreement between the two methods tended to occur when penguins appeared to turn round on the bridge, or were stationary towards the centre the bridge.

### Testing automatic selection of start and end points

In order to test effectiveness of the algorithm used to select the portion of the weighbridge data graph for calculation of penguin weight, 6480 graphs from the 2009–10 breeding season were examined manually. Appropriate start and end peaks and a direction were assigned to each graph. The mean weight values calculated from these start and end times were compared with those with start and end times calculated automatically. There was a strong correlation of 0.998 between the mean weight values calculated by both methods, with 67.5% of the mean weight values agreeing to within 10g and 98.5% to within 50g. Examination of graphs where there was larger disagreement showed that this was usually because more than one penguin crossed the bridge and the automatic method did not pick up when either the first penguin left the bridge, or the second penguin entered the bridge. Manually examining every crossing graph to identify the start and end points takes a significant amount of time meaning it is not a viable option for routine processing of the data. This test shows that the automatic method identifies appropriate start and end points for the majority of weighbridge crossings. Directions assigned manually agreed well with those assigned automatically with less than 0.5% crossings assigned a direction manually which disagreed with the one assigned automatically. The two automatic methods of assigning direction complemented one another as only 12 weighbridge crossings could not be assigned a direction with either method, and conflicting values were assigned for only 2 crossings.

### Comparison with manual penguin weights

**Fig 8:**
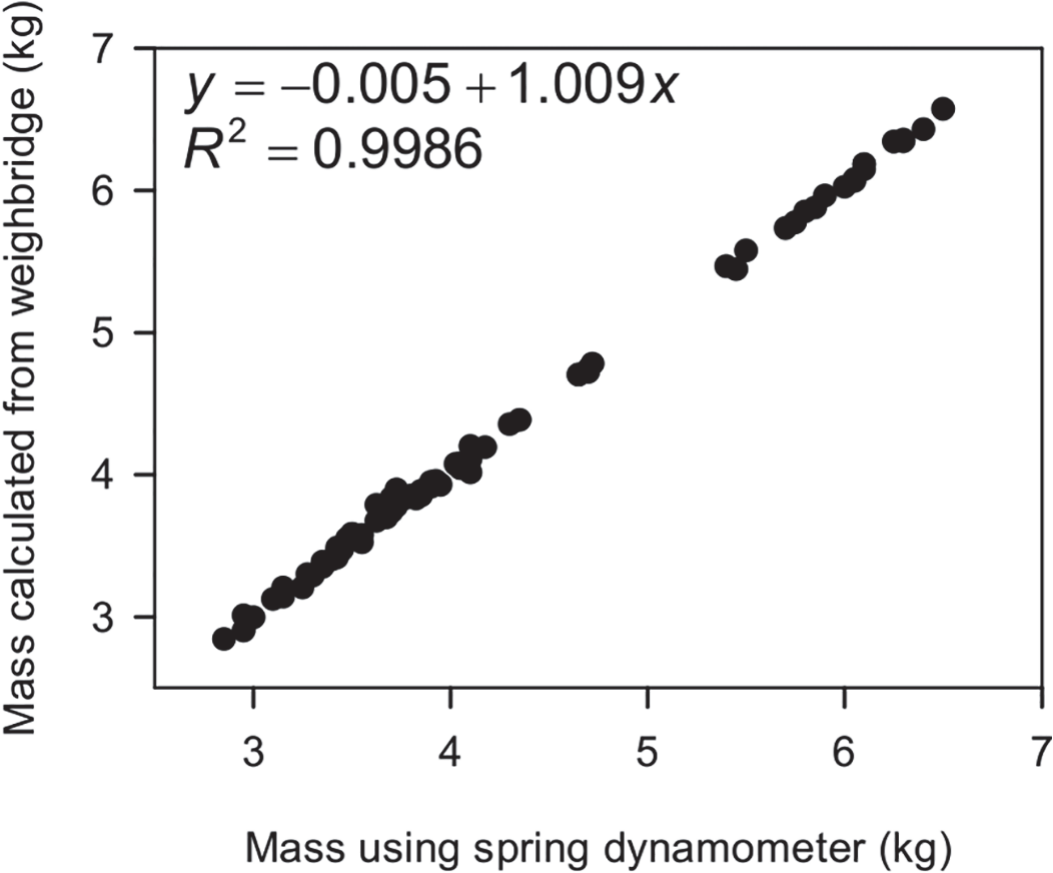
Dynamometer versus weighbridge weights. A comparison of weights calculated from weighbridge crossings with the mass of the same penguin obtained using a spring dynamometer once the penguin had crossed the bridge.

Each year a small number of penguins were caught once they had crossed the weighbridge, and weighed manually using a spring dynamometer. The manual weights and weights calculated from the weighbridge were compared. A comparison of dynamometer and weighbridge weights for 79 penguins from three seasons is shown in Fig 8. There is very strong agreement between the two methods across the whole range of possible penguin weights. The difference between the two methods ranged from 1 g to 169 g, with a mean of 42 g. As the spring dynamometer only record weights to the nearest 50 g, and can be affected by factors such as penguin movement in the restraining bag, wind and build up of dirt in the bag, then the significant agreement between manual and weighbridge weights suggests that we can have confidence that the weights calculated from the weighbridge files are indeed accurate.

### Comparison with other weighbridge systems

**Fig 9:**
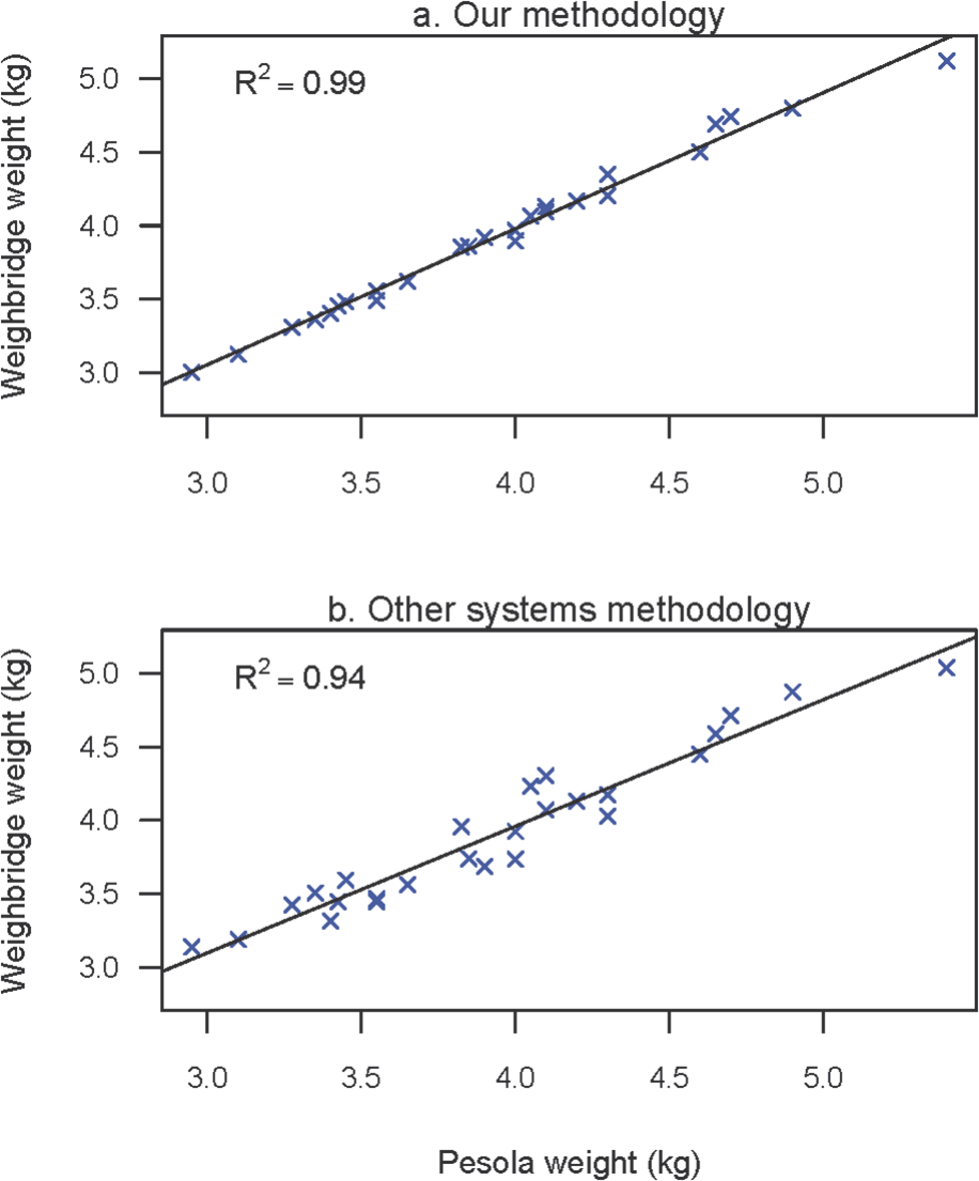
A comparison of weighbridge systems against manual weights. The top graph shows a regression of manual weights against weighbridge weights calculated using the methodology described in this paper, whereas the bottom graphs compares the manual weights with weights calculated using the methodology of other weighbridge systems.

Without running our weighbridge system in tandem with an existing system it is difficult to quantify what improvements in accuracy it makes. We have, however, attempted to process our data using similar methods to those used in other systems. This was done by taking 25 of the files for 2011–12 where we had both a manual and a weighbridge weight, reducing the sampling frequency of the file to 50s^-1^, calculating the offset using the regression method outlined in Robinson et al.[17], using the data from when the penguin first stepped on to the weighbridge until it stepped off and not calculating the velocity component. Of the 25 files tested the methodology described in this paper gave a weight that was closer to the dynamometer weight in 22 of the 25 files (Fig 9). Using our methodology the weighbridge weights were on average 0.04 kg different from the manual weights with a range of 0.01 to 0.1 kg (R^2^ = 0.99) whereas using the alternative method the weights were on average 0.12 kg different with a range of 0.01 to 0.27 kg (R^2^ = 0.94). This suggests therefore that our system is demonstrably more accurate than previously used systems.

### Data

**Fig 10:**
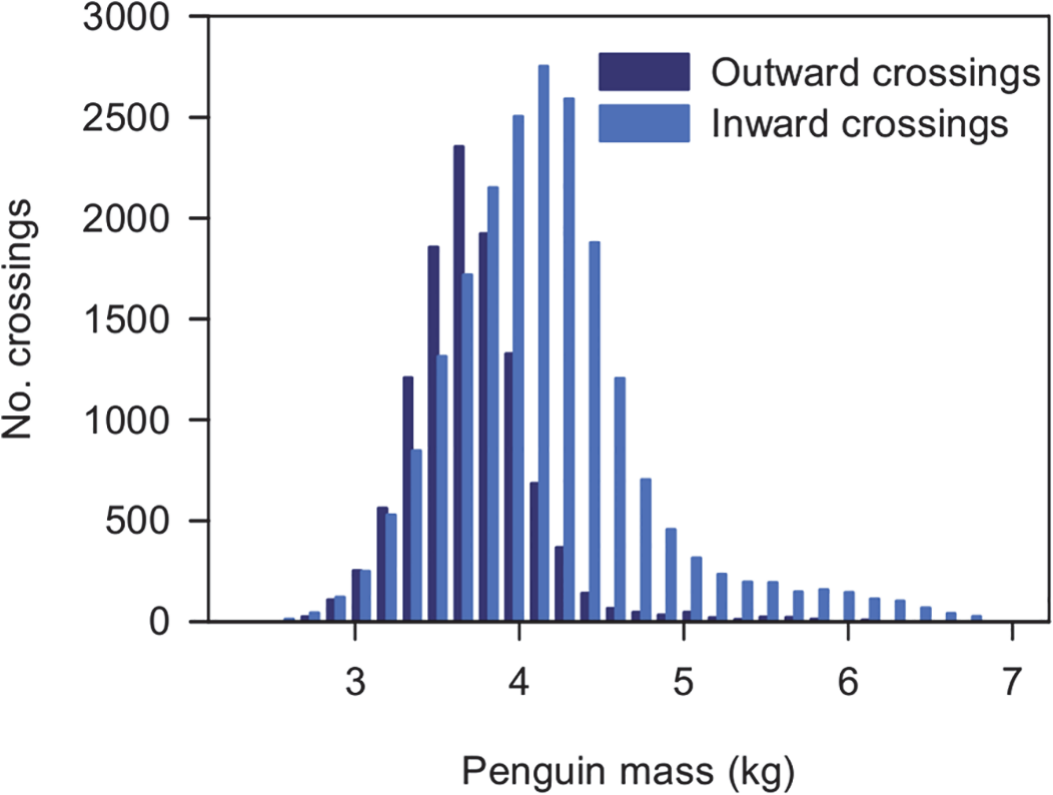
A histogram of penguin weights calculated from weighbridge crossing data for 2011–2012. A summary of the data from the 2011–2012 breeding season is shown. The weighbridge recorded 71429 files. Data are shown for 31997 crossings in total with the penguin weight calculated from at least 1.5 s on the bridge—11141 outward crossings and 20856 inward crossings.

A full analysis of the data from the weighbridge is currently in progress and will be reported in subsequent publications. A summary of the data from the 2011–2012 breeding season is shown in Fig 10 as a histogram of all the outward and inward crossings. Data are shown for 31997 crossings in total with the penguin weight calculated from at least 1.5 s on the bridge—11141 outward crossings and 20856 inward crossings. In total 813 different penguin RFID tags were recorded, ranging from 1 recorded crossing up to a maximum of 146 crossings with a mean of 47 crossings recorded per tagged penguin. Penguin tags are not always picked up by the RFID receiver—the likelihood of it being recorded may depend on the height and orientation of the implant as well as the number of birds on the weighbridge.

**Fig 11:**
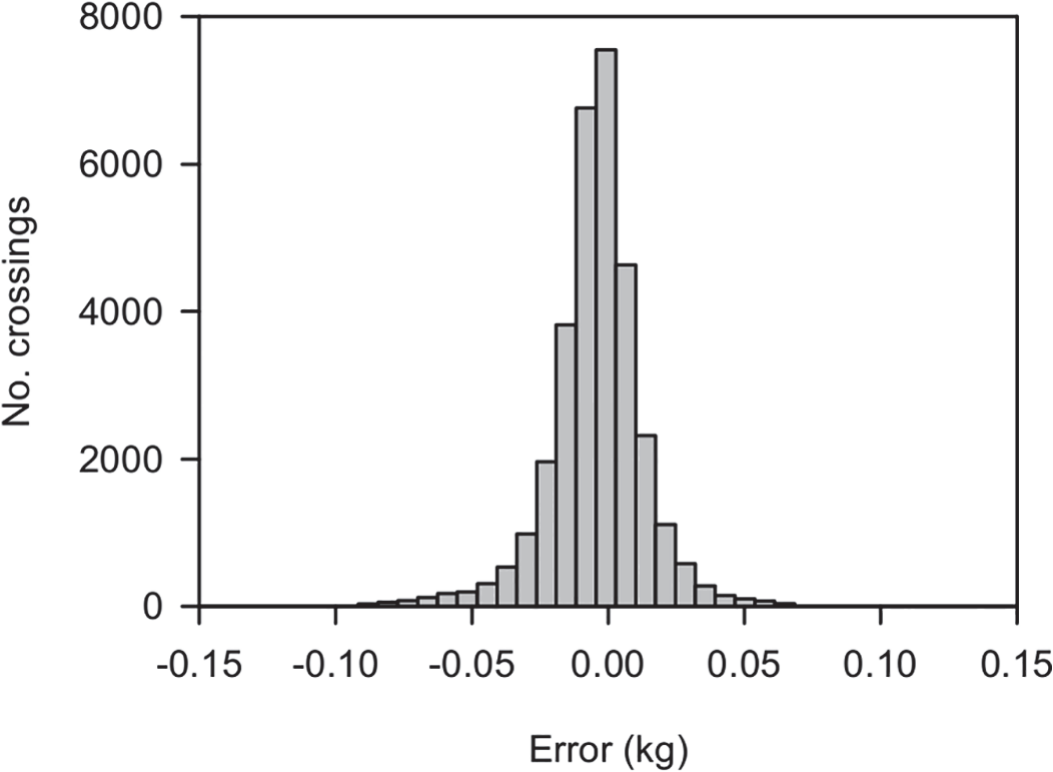
Weight difference between the first approximation of penguin weight and the second approximation.

A comparison was made of the estimated penguin weight from the first and second weight approximations to see how useful the second approximation was. A histogram of the calculated error for the 2011–2012 breeding season data is shown in Fig 11; 95% of crossings have an error of less than 40 g showing that the first approximation of weight is within the 1% level of accuracy required of the weighbridge for the vast majority of crossings.

## Discussion and Conclusion

This new design of penguin weighbridge has proven to be robust in the field—with very little loss of data at any point during four seasons of deployment to date. We are confident, from the tests that we have conducted, that the data it records allow very close approximations to penguin static weights to be made (Figs 10 and 11). Our design is significantly different to existing weighbridge systems in a number of important factors. We have demonstrated that it is necessary to minimise the measurement error of the system (Eq 2). This is done, firstly, by selecting carefully the start and end point of the integration interval used, and secondly, by using estimates of penguin velocity to calculate the error associated with our weight estimation so it can be removed. Existing weighbridge systems do neither of these, but adjust for error by calibrating with known static weights and by comparing weighbridge weights with those of penguins weighed using a spring balance (e.g., [1,13]). The latter method is potentially much less accurate as the adjustment is not tailored to individual crossings, as in our system. The sampling frequency (200s^-1^) of our system is several times greater than that used by other systems (e.g. 60s^-1^,[18]) which avoids distortions due to resonant frequency and collects more data when crossing times are short. We also report the resonance frequency of our weighbridge. This factor is important as penguins moving on a weighbridge cause oscillations which in turn affect the linearity of the results. By using a high resonant frequency this distortion is minimised. Previously published papers [17,18], do not provide data on the resonant frequency of their systems. A further advantage of our system is regular calculation of the zero offset. Some existing systems have measured this using static calibrations and then calculate daily drift rate using regression analysis [17]. Changes in zero offset, however, are due to build up of mud/snow/sand on the weighbridge and can change both upwards and downwards in a short space of time. These changes are unpredictable and can be significant. In order to minimise this issue we have designed our system to calculate zero offset for every crossing when a penguin leaves the bridge, before the data file finishes recording (Fig 6). This is possible for 60% of all files. For all other files we use the nearest available offset. We have demonstrated that our system, and the algorithms we use to calculate penguin mass, produce values that compare very closely with manual weights and are more accurate than mass values calculated without taking account all of the factors we have listed as important. This increase in accuracy is particularly important if the data are going to be used to estimate variables such as meal mass. Retention of all the raw data is also a significant advantage over previous types of weighbridge. It allows the raw data to be viewed by scientists to check for multiple birds and/or direction (e.g. Figs 6 and 7) and allows for alternative data processing algorithms to be used. Users of data with different research objectives may benefit from using different processing algorithms.

The data from this weighbridge will allow scientists to monitor the weight of individual penguins in a reasonably sized colony across a whole season and ultimately across multiple seasons. There are, however, a few issues that need to be borne in mind when analysing the data. Firstly there would appear to be many more crossings going in to the colony recorded than crossings going out of the colony. It is likely that this is due to the behaviour of the penguins. There is a tendency for the penguins to bunch up as they leave the colony at similar times in the morning, meaning that a lot of crossings get filtered out during the processing stage as weights cannot be calculated from crossings when there is not a long enough time with just one bird on the bridge. The penguins are more spaced out when returning to the colony after feeding increasing the number of single bird crossings, so less are filtered out during processing. Secondly, it is difficult to study the changes in weight of individual penguins in detail throughout a season, because there are some crossings where the RFID tag is not recorded and some crossings where the tag is recorded but a reliable weight cannot be estimated due to there being more than one penguin on the bridge. However, the large sample of penguins that have been tagged, and the good proportion of crossings for which weights are available, give a variety of options for studying factors such as: average penguin weights and average meal size at different points in the season, average trip lengths and differences in feeding behaviour between male and female penguins.
